# Inferring Geographic Coordinates of Origin for Europeans Using Small Panels of Ancestry Informative Markers

**DOI:** 10.1371/journal.pone.0011892

**Published:** 2010-08-18

**Authors:** Petros Drineas, Jamey Lewis, Peristera Paschou

**Affiliations:** 1 Department of Computer Science, Rensselaer Polytechnic Institute, Troy, New York, United States of America; 2 Department of Molecular Biology and Genetics, Democritus University of Thrace, Alexandroupoli, Greece; State University of New York College at Oneonta, United States of America

## Abstract

Recent large-scale studies of European populations have demonstrated the existence of population genetic structure within Europe and the potential to accurately infer individual ancestry when information from hundreds of thousands of genetic markers is used. In fact, when genomewide genetic variation of European populations is projected down to a two-dimensional Principal Components Analysis plot, a surprising correlation with actual geographic coordinates of self-reported ancestry has been reported. This substructure can hamper the search of susceptibility genes for common complex disorders leading to spurious correlations. The identification of genetic markers that can correct for population stratification becomes therefore of paramount importance. Analyzing 1,200 individuals from 11 populations genotyped for more than 500,000 SNPs (Population Reference Sample), we present a systematic exploration of the extent to which geographic coordinates of origin within Europe can be predicted, with small panels of SNPs. Markers are selected to correlate with the top principal components of the dataset, as we have previously demonstrated. Performing thorough cross-validation experiments we show that it is indeed possible to predict individual ancestry within Europe down to a few hundred kilometers from actual individual origin, using information from carefully selected panels of 500 or 1,000 SNPs. Furthermore, we show that these panels can be used to correctly assign the HapMap Phase 3 European populations to their geographic origin. The SNPs that we propose can prove extremely useful in a variety of different settings, such as stratification correction or genetic ancestry testing, and the study of the history of European populations.

## Introduction

The study of human population genetic structure and the selection of Ancestry Informative Markers (AIMs) have attracted considerable attention, mainly due to their implications for diverse areas of genetics and a variety of research scenarios, ranging from forensics to population genetics and medical genetics. Within the European continent, early studies of population genetic structure sought to address questions on the origin of different ethnic groups as well as the historic and genetic relationships among them. Indeed, studies of variation on the non-recombining portion of the Y chromosome supported the hypothesis of an initial settlement of Europe by Paleolithic hunter-gatherer communities, as well as the European re-colonization from glacial refugia in the South and, later, from a rapidly expanding farming population originating from Anatolia [1], [2], [3]. The advent of large-scale genotyping allowed us to further explore these hypotheses and also revealed the practical implications of identifying and understanding European population genetic structure. In the search of susceptibility genes for common complex disorders, it became evident that population stratification within Europe does exist and that it can lead to spurious results when coupled with phenotype correlations with geography [4], [5].

With the volume of rich genotypic data rapidly increasing, Principal Components Analysis (PCA) emerged as a powerful technique that can be used to summarize and process the vast amounts of available information. PCA is a linear dimensionality reduction technique that can effectively extract the fundamental structure of a dataset without any need for modeling of the data. It has been used to decompose the complex genetic structure of human populations [6], [7] and it can be successfully applied to infer genetic ancestry as well as substructure in a given sample [8], [9], [10], [11]. Furthermore, as we have recently described, PCA can be applied to identify AIMs, which in this case represent SNPs that are correlated with significant Principal Components (PCA Informative Markers - PCAIMs) [8], [9]. In fact, we demonstrated that small panels of such SNPs can successfully reproduce the structure of a dataset as identified by PCA, without any prior knowledge or hypothesis about on the origin of studied individuals or artificial assignment of individuals to pre-defined clusters [8], [9].

Leveraging the power of PCA, recent large-scale studies have allowed us to appreciate the fact that population genetic structure within Europe is discernable at a fine scale, when information from hundreds of thousands of genetic markers that span the entire genome is used [12], [13], [14], [15], [16], [17]. A number of recent studies analyzed thousands of individuals across Europe using information from genomewide genotypes and showed that the top two principal components capture a significant amount of variation across European populations [14], [15], [16], [17]. These studies also demonstrated a surprising correlation of the top two principal components with longitude and latitude by showing that the two-dimensional PCA plot of genomewide genotypes yields patterns that are reminiscent of the geographic map of Europe [14], [15]. This information can subsequently be used to place individuals within a few hundred kilometers of their reported origin [15].

In our work here we explore the extent to which geographic coordinates within Europe can be predicted based solely on information from small subsets of genetic markers. We investigate a subset of the Population Reference Sample (POPRES), comprising of 1,200 individuals from 11 populations [15], [18]. Using algorithmic tools that we have previously described, we select small subsets of Single Nucleotide Polymoprhisms (SNPs) that correlate well with population structure, as captured by PCA [8], [9]. This is the first study to systematically explore this question as a classification problem by performing thorough cross-validation experiments in order to assign individuals of “unknown” origin to specific geographic locations in Europe.

## Methods

### POPRES and HapMap Phase 3 Europeans

We analyzed a subset of the Population Reference Sample (POPRES) as described in [15] consisting of 1,387 samples. We focused on populations with at least 40 available samples, thus retaining 1,200 individuals from 11 populations. These samples have been genotyped using the Affymetrix 500K array. We kept 447,212 autosomal SNPs after removing markers with 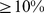 missing entries. We also analyzed the two European HapMap Phase 3 populations: CEPH Europeans (CEU) and Tuscans (TSI).

### Selecting PCA-Informative Markers (PCAIMs)

We computed PCA scores for each SNP using the algorithm of [8] and we selected the SNPs with the highest scores (PCAIMs). In order to remove redundancy from the selected set of markers, we employed a method that we have previously described in [9].

### Prediction of geographic coordinates via Nearest Neighbors

We used as ground-truth geographic coordinates the ones provided in [15], which typically place a sample to the capital city of his/her country of origin. In order to predict coordinates for unassigned individuals, we used a simple Nearest Neighbors (NN) approach. 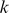-NN algorithms first compute the distance of the new sample from the 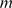 individuals in the database and then identify the 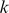 nearest neighbors of the new sample. In order to predict the coordinates of the new sample, we simply compute the average of the coordinates of its 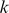 nearest neighbors (we set 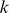 to ten). In all our experiments our distance metric was the standard Euclidean distance. The distance was computed on the projection of the genotypic data on their top two principal components. We experimented with different values of 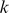 (the number of nearest neighbors) ranging from ten up to 20 in increments of one, but we did not observe a consistent advantage in using any value above ten. Similarly, we experimented with various schemes using weighted averages of the coordinates of the top 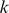 nearest neighbors (for example, the contribution of the coordinates of a neighbor to the final prediction could be weighted by – some power – of the inverse of its distance to the new sample); once more, we did not observe a consistent advantage in using such schemes. While we can not rule out that more advanced classification methodologies and/or better distance metrics might be applicable in order to improve prediction accuracy, it is quite interesting and exciting that standard, simple methods are quite accurate and useful.

### Crossvalidation experiments

We ran two different crossvalidation experiments.

#### Leave-one-out crossvalidation

We cross-validated a total of 1,200 individuals from the eleven populations in our dataset that had more than 40 samples. In each of the 1,200 repetitions of this experiment, we left out one individual (test set) and used the remaining individuals as the training set. We then used the training set individuals to compute panels of AIMs of various sizes (PCAIMs with redundancy removal) and then we employed our NN algorithm in order to predict the coordinates of origin of the test set individual.

#### HapMap Phase 3 data

Our second cross-validation experiment uses as training set the POPRES samples and as the test set the HapMap Phase 3 CEU and TSI populations. While extracting genotypes for our POPRES-based panels from the HapMap data we excluded individuals from the HapMap populations that had more than 10% missing entries on our panels.

More details on data encoding, PCA, and our SNP selection procedures are available in Methods S1.

## Results

### Ancestry inference using all available SNPs

Our first experiment measured the prediction accuracy of our NN algorithm using all available SNPs. The average latitudinal error is 0.99 degrees (a very small deviation) and the average longitudinal error is 2.52 degrees. Interestingly, we get a better prediction of the North-North West to South-South East axis as opposed to the East to West axis. It is also worth noting that the largest average error was in the German samples and that the most accurately predicted populations were the Southern European and Irish ones. In our supporting online material (http://www.cs.rpi.edu/~drinep/POPRESAIMS, Text S1) we included plots for each of the eleven largest populations in our sample showing the mean and the standard deviation for each of the predicted populations.

### Validation experiments using small panels of PCAIMs

As a first step, in order to verify our methodology, we attempted to evaluate whether there exist small panels of AIMs that could accurately reproduce the results of coordinate prediction using all 450K available markers. We started by selecting the top 5,000 PCA-Informative markers using two significant principal components. We then removed redundant markers using the algorithm of [9] and constructed three different panels of PCAIMs: P1 containing 500 markers, P2 containing 800 markers, and P3 containing 1,000 markers. The goal of this experiment is to illustrate that a relatively small (less than .2% of the total number of available SNPs), albeit carefully selected, set of markers suffices for ancestry inference. Indeed, Table 1 indicates the performance of our three PCAIMs panels. The performance of all panels is quite satisfactory, with the largest panel typically being no more than two times worse than the performance of all 450K markers. Especially in countries where the error was large even using all 450K markers (for example, Germany), our panels perform almost as well as the full set of markers. It is important to emphasize that this experiment simply illustrates the fact that the information contained in the full set of 450K markers can be efficiently summarized using only a small number of carefully selected representative AIMs. However, we have not yet selected AIMs in the setting of a true cross-validation experiment. Indeed, the AIMs selected above were the result of processing the full dataset, without splitting it in training and test sets first; this will be done in our next experiment. Finally, we note that detailed lists of all panels (P1, P2, and P3) appear in the online material accompanying this work (http://www.cs.rpi.edu/~drinep/POPRESAIMS, Text S1).

**Table 1:** Latitudinal and longitudinal errors of our full leave-one-out validation experiment on 1,200 samples from 11 populations.

Population	Latitudinal Error			Longitudinal Error		
	P1	P2	P3	P1	P2	P3
Belgium (43)	2.24 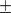 1.75	2.33 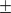 1.65	2.43 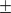 1.38	2.89 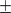 2.40	2.94 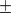 2.10	3.16 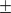 2.21
France (91)	2.98 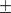 1.93	2.57 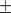 2.00	2.33 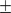 1.83	3.44 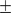 3.02	3.55 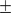 2.77	3.43 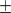 2.53
Germany (71)	2.60 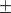 1.96	2.23 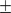 1.76	2.38 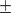 1.80	5.71 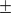 3.62	5.66 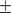 3.37	5.38 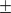 3.97
Ireland (61)	0.84 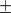 1.02	0.65 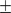 0.76	0.49 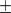 0.58	6.59 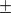 3.67	5.36 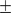 3.48	4.88 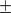 2.82
Italy (219)	0.89 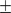 1.18	0.88 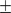 1.16	0.81 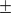 1.12	3.57 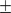 3.97	2.84 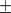 3.61	2.63 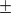 3.68
Portugal (128)	1.73 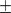 1.64	1.32 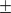 1.64	1.11 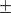 1.52	5.99 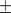 4.44	4.68 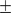 3.91	4.02 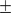 3.37
Serbia (44)	1.35 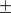 0.74	1.17 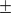 0.72	0.98 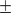 0.70	3.87 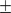 3.38	2.84 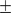 2.72	2.22 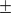 2.35
Spain (136)	1.63 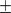 1.83	1.00 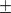 1.32	0.93 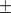 1.34	3.18 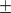 2.77	2.41 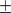 2.23	2.54 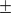 2.40
SwissF (125)	2.18 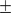 1.67	1.81 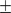 1.42	1.64 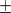 1.33	3.29 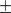 2.62	2.74 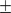 2.46	2.36 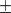 2.07
SwissG (84)	1.98 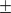 1.53	1.49 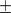 1.57	1.36 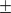 1.43	4.10 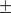 2.44	3.67 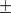 2.35	3.27 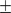 2.20
UK (200)	2.00 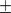 2.06	1.59 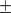 1.81	1.37 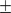 1.72	4.81 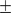 3.96	4.01 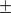 3.57	3.62 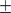 3.47

Results are reported for three panel sizes (P1:500 SNPs, P2:800 SNPs, P3:1000 SNPs). For each panel size and for each population we report the average error and the standard deviation.

### Leave-one-out cross-validation experiment

**Figure 1:**
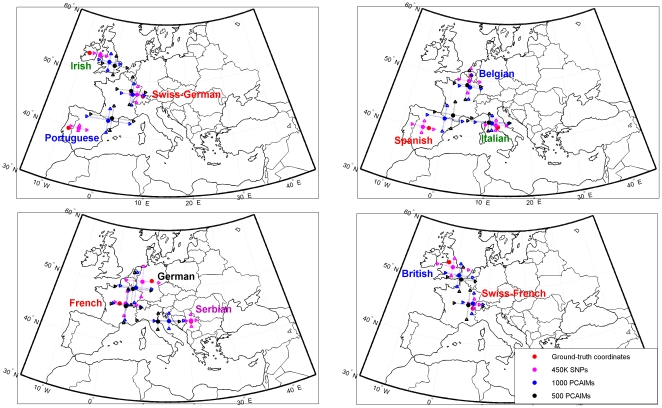
Complete leave-one-out cross-validation experiment. Predicted ancestry location (and standard deviation) of studied individuals using PCAIM panels of different sizes. Reference geographic locations associated with each population were assigned using the central point of the geographic area of the country as in Novembre et al. [15].

We performed 1,200 splits of data, where in each split we constructed a test set consisting of one individual and the remaining individuals were used as a training set in order to select PCAIMs and predict the coordinates of the test set sample. Figure 1 and Table 2 summarize the performance of our PCAIM panels over all 1,200 individuals in all test sets. The overall performance of our approach using even small panels of PCAIMs is quite remarkable for almost all populations. Especially in terms of latitude, the average error never exceeds three degrees using our largest panel. Even with the smallest panel of 500 SNPs we show satisfactory prediction accuracy that actually exceeds the three degree error threshold only for the Spanish and Portuguese populations. With respect to the more challenging longitudinal predictions, we observe that they are somewhat worse when compared with the performance of all 450K SNPs. In particular, the error in the Serbian population increases to an average of 5.6 degrees (as opposed to less than one degree using all SNPs). Similar increases of a factor of two are observed in the Irish and Italian populations, while the Portuguese population suffers a three-fold loss in accuracy. This illustrates that the East-West axis in Europe is somewhat harder to predict with high accuracy using a small number of SNPs, necessitating either larger panels of SNPs or more advanced methods.

**Table 2:** Latitudinal and longitudinal errors of our full leave-one-out crossvalidation experiment on 1,200 samples from 11 populations.

Population	Latitudinal Error			Longitudinal Error		
	P1	P2	P3	P1	P2	P3
Belgium (43)	1.98 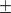 1.66	1.94 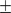 1.68	1.87 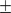 1.59	3.13 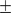 2.82	2.56 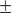 2.34	2.60 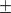 2.36
France (89)	2.12 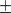 1.53	1.86 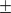 1.43	1.80 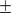 1.40	3.27 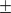 2.63	2.91 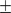 2.20	2.78 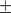 2.58
Germany (71)	2.25 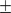 1.60	2.12 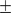 1.60	1.92 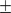 1.37	5.25 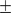 4.46	4.73 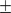 3.62	4.61 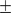 3.80
Ireland (61)	1.49 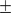 1.46	0.98 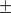 1.28	0.98 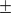 1.35	4.77 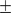 3.37	3.58 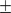 2.92	3.49 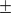 2.36
Italy (219)	1.02 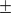 1.04	0.86 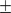 0.84	0.76 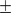 0.81	3.63 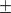 3.83	2.69 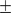 3.24	2.42 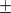 2.96
Portugal (128)	2.89 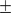 2.10	2.63 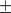 2.10	2.41 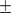 2.00	7.27 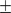 5.14	6.89 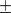 5.07	6.47 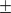 4.91
Serbia (44)	1.04 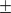 0.64	1.10 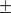 0.73	0.93 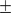 0.72	8.28 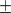 5.23	6.25 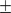 3.78	5.81 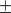 3.41
Spain (136)	3.21 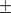 2.24	2.55 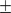 2.04	2.35 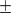 2.07	6.79 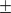 4.33	5.64 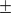 4.54	5.09 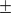 4.01
Swiss French (125)	1.88 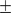 1.57	1.46 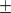 1.28	1.37 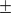 1.09	2.97 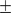 2.25	2.44 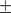 2.09	2.00 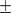 1.65
Swiss German (84)	2.05 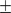 1.70	1.57 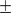 1.37	1.40 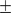 1.20	3.15 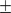 2.73	2.65 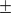 2.34	2.41 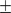 2.40
UK (200)	2.21 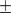 1.79	1.82 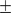 1.61	1.53 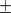 1.48	4.55 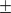 3.32	4.01 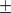 3.12	3.59 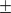 2.94

Results are reported for three panel sizes (P1:500 SNPs, P2:800 SNPs, P3:1000 SNPs). For each panel size and for each population we report the average error and the standard deviation.

### Predicting coordinates for the TSI and CEU populations

**Figure 2:**
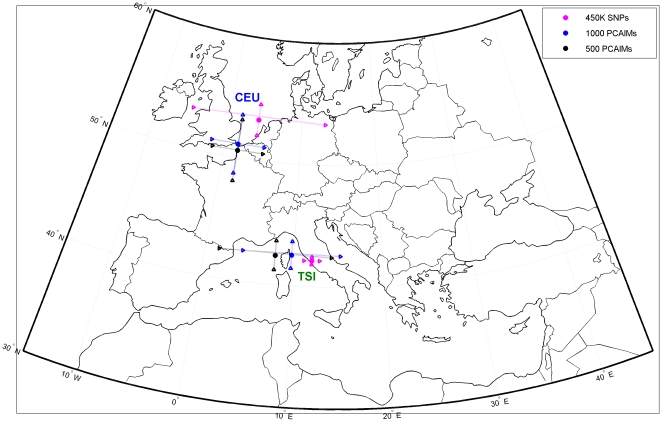
Cross-validation of our PCAIM panels for the classification of the European HapMap Phase 3 populations (CEPH Europeans-CEU and Tuscan Italian-TSI). Notice that out of the 1,000 SNPs of the first PCAIM panel, 994 SNPs were found in the HapMap CEU data and 927 in the HapMap TSI data. Similarly, out of the 500 SNPs of the second PCAIM panel, 496 SNPs were found in the HapMap CEU data and 459 in the HapMap TSI data.

In our second cross-validation experiment we evaluated the performance of the SNP panels derived using the full POPRES data as training set in order to classify individuals from the two European HapMap Phase 3 populations (CEPH Europeans-CEU and Tuscan Italian-TSI). We extracted the genotypes corresponding to CEU and TSI individuals from HapMap release 27 (built 36) raw data and then used our NN prediction algorithm to predict coordinates for the samples using all available SNPs as well as panels P1, P2, and P3. For the TSI and CEU samples, we chose to use as ground truth coordinates our predictions using all 450K SNP panels. Figure 2 illustrates the location of the CEU and TSI populations in the European map, with the red circle denoting the average CEU or TSI subject and the horizontal red lines illustrating the standard deviation in latitude and longitude. The red x and the blue x (along with the corresponding lines) illustrate our coordinate predictions using the 1000 and 500 SNP panels that were selected in the POPRES data. Note that not all SNPs of those panels were present in the HapMap data; for example, for the CEU samples, we found 994 SNPs from P3 in the HapMap data, and 496 SNPs from P1. (These numbers were slightly smaller – 927 and 459 respectively – for the TSI data.) Both our panels do a good job of predicting the location of CEU and TSI samples. In the TSI samples there is essentially no error in the North-South axis, but we are off by a few degrees in the East-West axis using our largest panel. For the CEU data, both latitudinal and longitudinal predictions are off by only a few degrees. In Figures S1 and S2 we show histograms of the (latitudinal and longitudinal) errors for our CEU and TSI samples using our 1,000 SNP panel. These figures highlight that two thirds of the samples are very accurately predicted (with an error of two degrees at most in terms of latitude and eight degrees in term of longitude), but there also exist some isolated samples that are quite inaccurately predicted; these samples increase somewhat disproportionately the average prediction error and its standard deviation. This is very obvious in the case of the TSI samples, where – in terms of longitude – five samples have 18 degrees of error (they had their nearest neighbors in the Spanish and Portuguese populations) thus considerably driving up the error, while over 60 samples had less than three degrees of longitudinal error.

## Discussion

This study is a comprehensive investigation of the possibility to recover geographic coordinates of individual ancestry within Europe based solely on information from carefully selected panels of genetic markers. Analyzing 1,200 individuals from 11 European populations and more than 440,000 SNPs, we show that it is indeed possible to predict individual ancestry within Europe down to a few hundred kilometers from the place of origin, using information from relatively small, albeit carefully selected, subsets of SNPs. Importantly, our findings are supported by thorough cross-validation experiments, both on the analyzed subset of the POPRES dataset [15], [18] and the European HapMap populations. More than 1,200 SVDs for large matrices were computed, which, however, took only two weeks to run on commodity hardware, thanks to the efficient algorithms that we use. Interestingly, within Europe, individual origin seems much easier to predict along the North to South axis than along the East to West axis. This could indicate increased gene flow along the latter axis.

The reduction in the number of markers needed for ancestry inference is made possible through the use of our PCA-based method for the selection of AIMs and our redundancy removal algorithm. Different metrics have been proposed in order to select AIMs, most of which, such as 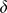 or Wright's 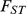 rely on the maximization of allele frequency differences between pre-defined populations [19], [20], [21], [22], [23]. A closely correlated measure, Informativeness for assignment (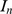) as defined by Rosenberg et al. [24] computes a mutual information based metric on allele frequencies. Our algorithm on the other hand [8], [9] does not rely on prior hypotheses about individual ancestry and is naturally coupled with other PCA-based algorithms, such as PCA-based stratification correction methods and the ancestry inference techniques that we describe here. Furthermore, as we have also demonstrated, the performance of our method for AIM selection is comparable or even superior, in some cases, to that of the metric of 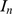 [8], [9].

Recent studies have underlined the existence of population substructure within Europe and a few of them have also explored the potential to uncover individual ancestry based on subsets of selected AIMs [9], [11], [10], [14], [15], [16], [17]. Heath et al. [16] investigated a panel of 391 PC correlated SNPs for ancestry inference in a sample of 6,000 individuals from across Europe. They showed some degree of correlation between predicted ancestry and ground truth, however, since this was not their main goal, they did not attempt cross-validation of this marker set. McEvoy et al. [17] focused on Northern European ancestry, studying a genomewide dataset of 2,099 individuals from eight populations of Northern European origin (including the admixed populations of European Australian and American individuals). They identified panels of AIMs based on the 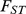 measure. Again, individual PC scores, in the studied Northern European populations, especially using the larger panels, were significantly correlated to PC scores using the full dataset [17]. Finally, Tian et al. [25], focused on AIMs selection for population differentiation along the North to South axis, by selecting 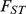-based SNPs for differentiation of Northern versus Southern European populations. However, that study focused on a relatively small sample of distinct European populations with a small number of samples for most populations. Here, we expand these studies, by offering SNP panels for ancestry inference and stratification correction, based on the largest publicly available dataset for European population structure.

The SNPs that we propose here as ancestry informative for European populations, can prove extremely useful for stratification correction in studies seeking to identify etiological genes for common complex disorders, when candidate susceptibility loci are targeted in larger samples, following an initial genome scan. In such cases, the inclusion of AIMs genotyping is essential, especially if underlying population structure related to the phenotype is suspected. Furthermore, these SNPs warrant further study, as they could underlie observed differences in disease frequency across Europe (for instance, the well-noted North to South gradient in the incidence of autoimmune disorders, such as type 1 diabetes [26]. Although, such SNPs could have reached their population differentiating frequencies and patterns, due to demographic factors, it is possible that natural selection has operated on them. In fact, the top SNPs on our lists reside in the lactase gene region which is well known to have undergone a recent selective sweep [27]. Further work will shed light into the relative contribution of migration, and drift versus natural selection in shaping the patterns of genomewide variation in the European population.

## Supporting Information

Figure S1 Distribution of the latitudinal (panel A) and longitudinal error (panel B) when using a panel of 994 SNPs selected on the POPRES samples to predict the coordinates of origin of the HapMap Phase 3 CEU samples. We consider as ground truth for the CEU samples our predictions using all 450K available SNPs.
(0.13 MB PDF)

Figure S2 Distribution of the latitudinal (panel A) and longitudinal error (panel B) when using a panel of 927 SNPs selected on the POPRES samples to predict the coordinates of origin of the HapMap Phase 3 TSI samples. We consider as ground truth for the TSI samples our predictions using all 450K available SNPs.
(0.12 MB PDF)

Methods S1 (0.04 MB PDF)

Text S1 (0.01 MB PDF)

## References

[1] Belle E, Landry P, Barboujani G (2006). Origins and evolution of the Europeans' genome: Evidence from multiple microsatellite loci.. Proc Biol Sci.

[2] Torroni A, Bandelt H, Macaulay V, Richards M, Cruciani F (2001). A signal, from human mtDNA, of postglacial recolonization in Europe.. Am J Hum Genet.

[3] Chikhi L, Nichols RA, Barbujani G, Beaumont MA (2002). Y genetic data support the Neolithic demic diffusion model.. Proc Natl Acad Sci U S A.

[4] Marchini J, Cardon L, Phillips M, Donnelly P (2004). The effects of human population structure on large genetic association studies.. Nat Genet.

[5] Campbell C, Ogburn E, Lunetta K, Lyon H, Freedman M (2005). Demonstrating stratification in a European American population.. Nat Genet.

[6] Li JZ, Absher DM, Tang H, Southwick AM, Casto AM (2008). Worldwide human relationships inferred from genome-wide patterns of variation.. Science.

[7] Biswas S, Scheinfeldt LB, Akey JM (2009). Genome-wide insights into the patterns and determinants of fine-scale population structure in humans.. Am J Hum Genet.

[8] Paschou P, Ziv E, Burchard EG, Choudhry S, Rodriguez-Cintron W (2007). PCA-correlated SNPs for structure identification in worldwide human populations.. PLoS Genet.

[9] Paschou P, Drineas P, Lewis J, Nievergelt CM, Nickerson DA (2008). Tracing sub-structure in the European American population with PCA-informative markers.. PLoS Genet.

[10] Price A, Butler J, Patterson N, Capelli C, Pascali V (2008). Discerning the ancestry of European Americans in genetic association studies.. PLoS Genet.

[11] Tian C, Plenge R, Ransom M, Lee A, Villoslada P (2008). Analysis and application of European genetic substructure using 300 K SNP information.. PLoS Genet.

[12] Seldin M, Shigeta R, Villoslada P, Selmi C, Tuomilehto J (2006). European population substructure: clustering of northern and southern populations.. PLoS Genet.

[13] Bauchet M, McEvoy B, Pearson L, Quillen E, Sarkisian T (2007). Measuring European Population Stratification with Microarray Genotype Data.. Am J Hum Genet.

[14] Lao O, Lu TT, Nothnagel M, Junge O, Freitag-Wolf S (2008). Correlation between genetic and geographic structure in Europe.. Curr Biol.

[15] Novembre J, Johnson T, Bryc K, Kutalik Z, Boyko AR (2008). Genes mirror geography within Europe.. Nature.

[16] Heath SC, Gut IG, Brennan P, McKay JD, Bencko V (2008). Investigation of the fine structure of European populations with applications to disease association studies.. Eur J Hum Genet.

[17] McEvoy BP, Montgomery GW, McRae AF, Ripatti S, Perola M (2009). Geographical structure and differential natural selection among North European populations.. Genome Res.

[18] Nelson MR, Bryc K, King KS, Indap A, Boyko AR (2008). The Population Reference Sample, POPRES: a resource for population, disease, and pharmacological genetics research.. Am J Hum Genet.

[19] Parra E, Marcini A, Akey J, Martinson J, Batzer M (1998). Estimating African American admixture proportions by use of population-specific alleles.. Am J Hum Genet.

[20] Collins-Schramm H, Phillips C, Operario D, Lee J, Weber J (2002). Ethnic-difference markers for use in mapping by admixture linkage disequilibrium.. Am J Hum Genet.

[21] Dean M, Stephens J, Winkler C, Lomb D, Ramsburg M (1994). Polymorphic admixture typing in human ethnic populations.. Am J Hum Genet.

[22] Wright S (1951). The genetical structure of populations.. Ann Eugen.

[23] McKeigue P (1998). Mapping genes that underlie ethnic differences in disease risk: methods for detecting linkage in admixed populations, by conditioning on parental admixture.. Am J Hum Genet.

[24] Rosenberg N, Li L, Ward R, Pritchard J (2003). Informativeness of genetic markers for inference of ancestry.. Am J Hum Genet.

[25] Tian C, Kosoy R, Nassir R, Lee A, Villoslada P (2009). European Population Genetic Substructure: Further Definition of Ancestry Informative Markers for Distinguishing among Diverse European Ethnic Groups... Mol Med.

[26] Bach J (2002). The effect of infections on susceptibility to autoimmune and allergic diseases... N Engl J Med.

[27] Sabeti PC, Varilly P, Fry B, Lohmueller J, Hostetter E (2007). Genome-wide detection and characterization of positive selection in human populations.. Nature.

