# Supplementary figures and images for: Inferring Geographic Coordinates of Origin for Europeans Using Small Panels of Ancestry Informative Markers
Source: PLoS One. 2010 Aug 18;5(8):e11892. doi: 10.1371/journal.pone.0011892 (PMC2923600; doi:10.1371/journal.pone.0011892)

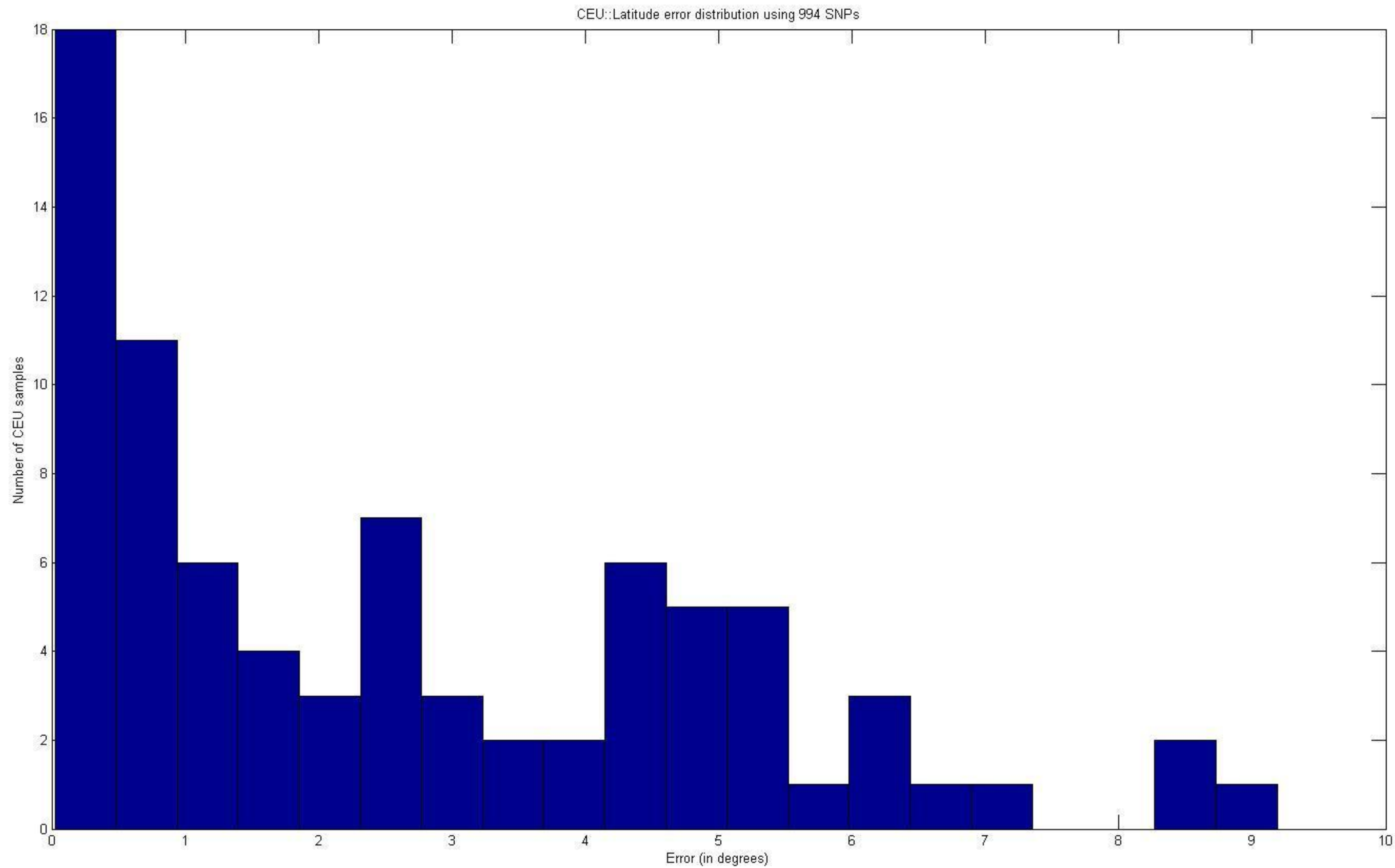

**Supplementary Figure 1A**

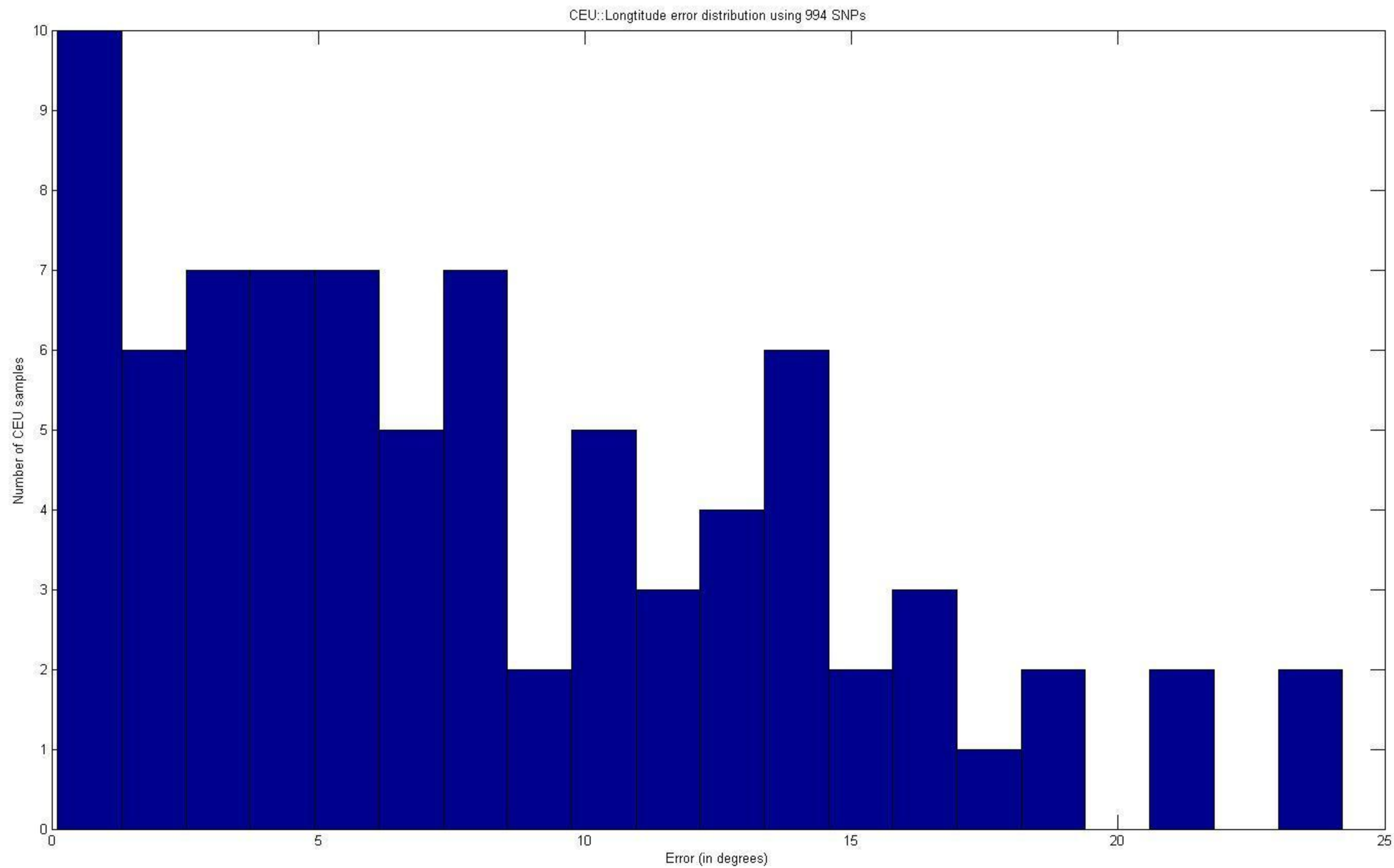

**Supplementary Figure 1B**

Supplement: Figure S1 — Distribution of the latitudinal (panel A) and longitudinal error (panel B) when using a panel of 994 SNPs selected on the POPRES samples to predict the coordinates of origin of the HapMap Phase 3 CEU samples. We consider as ground truth for the CEU samples our predictions using all 450K available SNPs. (0.13 MB PDF) [file pone.0011892.s001.pdf]

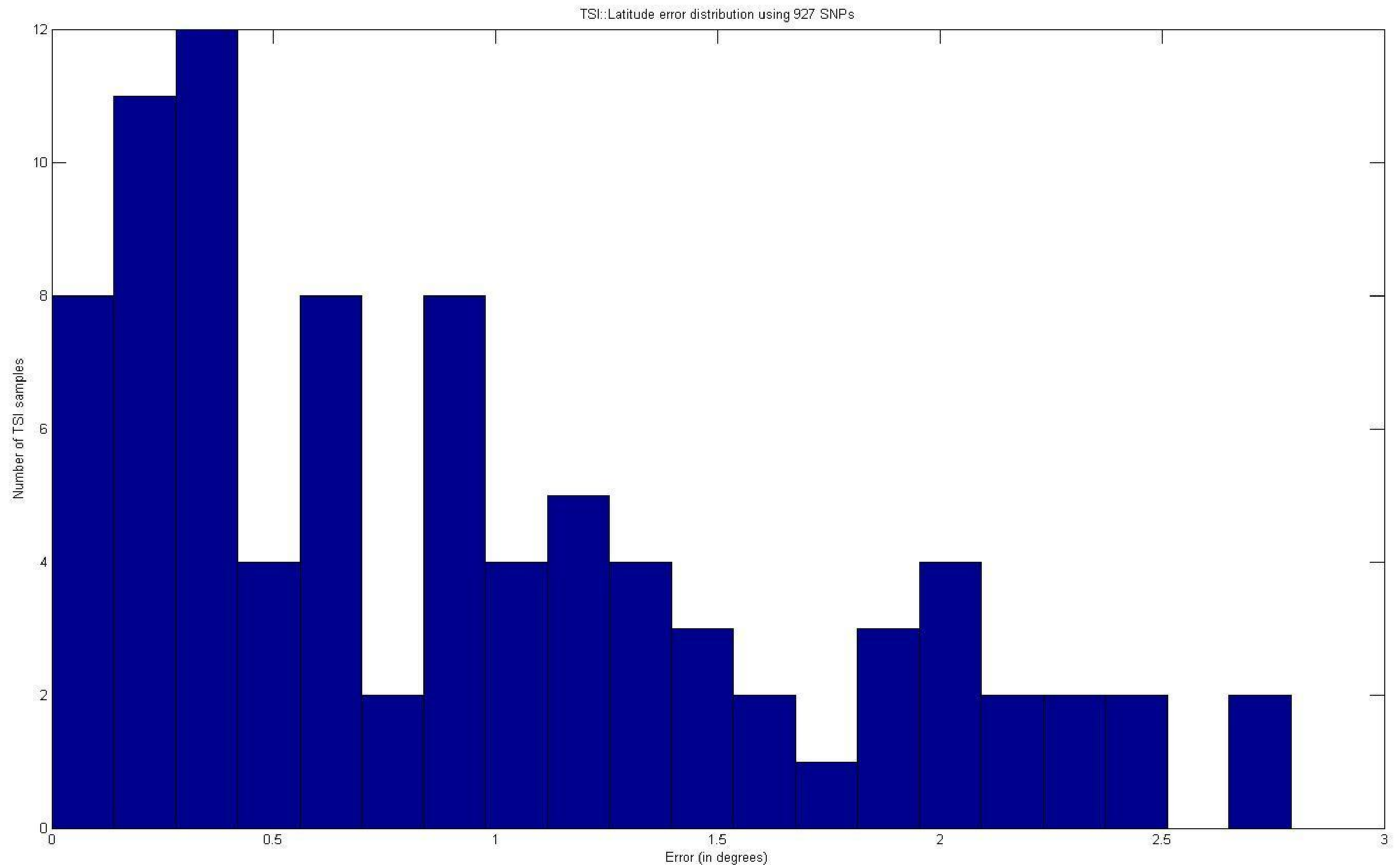

**Supplementary Figure 2A**

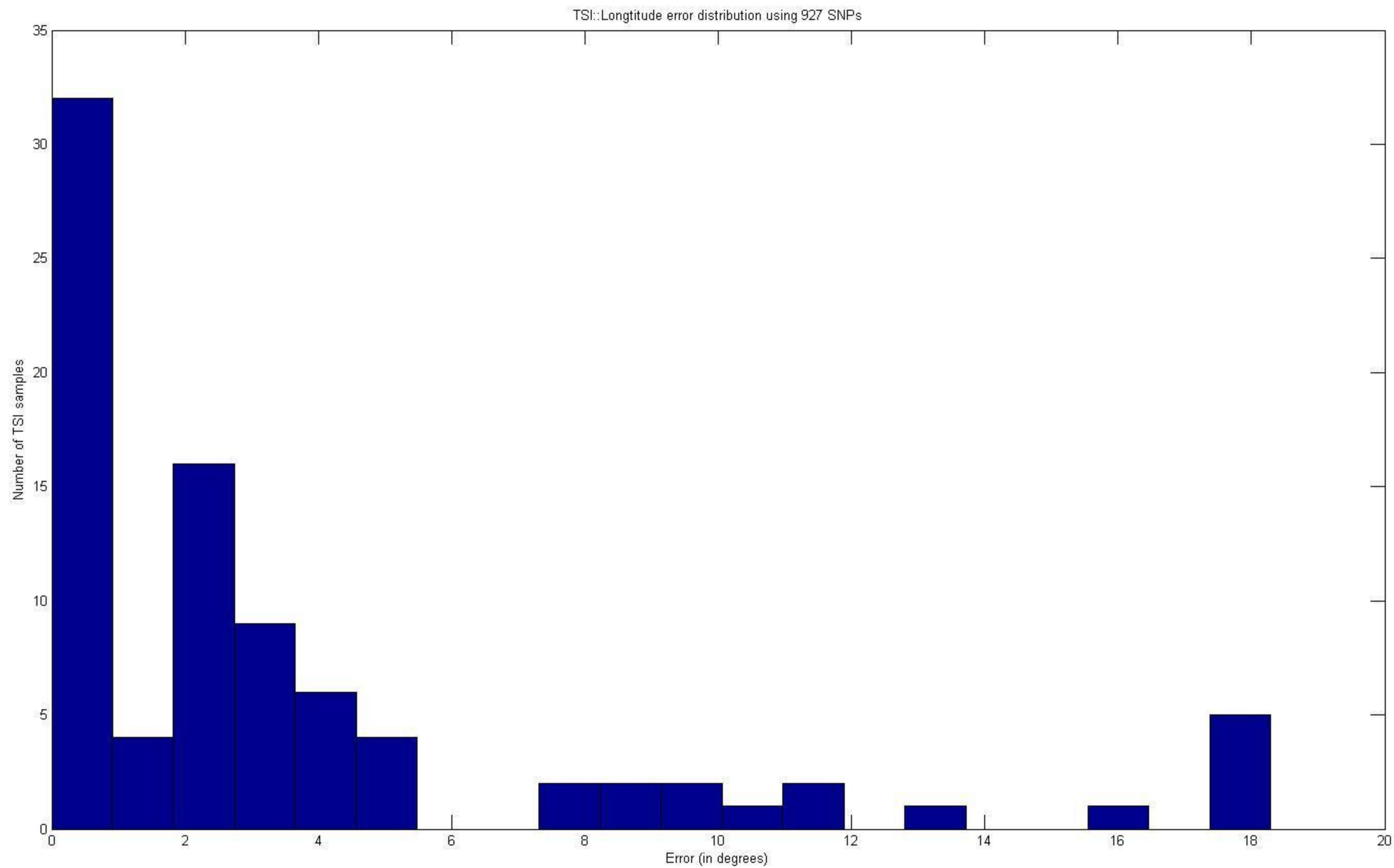

**Supplementary Figure 2B**

Supplement: Figure S2 — Distribution of the latitudinal (panel A) and longitudinal error (panel B) when using a panel of 927 SNPs selected on the POPRES samples to predict the coordinates of origin of the HapMap Phase 3 TSI samples. We consider as ground truth for the TSI samples our predictions using all 450K available SNPs. (0.12 MB PDF) [file pone.0011892.s002.pdf]
