# Supplementary material for: Inferring Geographic Coordinates of Origin for Europeans Using Small Panels of Ancestry Informative Markers
Source: PLoS One. 2010 Aug 18;5(8):e11892. doi: 10.1371/journal.pone.0011892 (PMC2923600; doi:10.1371/journal.pone.0011892)
Supplement: Methods S1 — (0.04 MB PDF) [file pone.0011892.s003.pdf]

Supplementary Methods

The data and the selection of AIMs

We studied a previously described dataset of 1385 individuals from 37 European populations [1]. (Actually, the dataset studied in [1] included 1387 samples; however, in the latest release two samples were omitted from the dataset due to privacy concerns and thus are not included in our study.) These samples are a subset of the Population Reference Sample (POPRES) [2] and were selected using stringent criteria to guarantee their European ancestry; see [1] for details. The samples were genotyped on approximately $450,000$ SNPs and we kept $447,212$ SNPs, after removing markers with more than 10% missing entries. Unlike [1] we decided to retain all SNPs, even those that are in high LD with each other. The reason behind our decision is two-fold: first, [1] removed a large number of markers to avoid artifacts that might be due to genomic regions that exhibit high LD in the results of Principal Components Analysis. However, since both [1] and [3] convincingly argue that PCA does reproduce geographic structure, we do not need to omit any markers in this work. It is worth noting that the correlation coefficient between the top two principal components using all available markers and the top two principal components using only the markers selected in [1] is very high (above 0.975). Second, [1] omitted genomic regions such as the one surrounding the LCT gene in order to avoid confounding the PCA results. However, for our purposes, those regions are particularly important since they correlate (and predict) well the north-to-south European axis. As a second dataset we also studied SNPs for the selected ancestry informative panels from the HapMap Phase 3 data on the CEPH European (CEU) and the Tuscan Italian population (TSI) [4, 5, 6]. For both datasets we only considered SNPs on autosomal chromosomes in our analysis.

For more details on encoding the data numerically in order to apply the Singular Value Decomposition (SVD) and Principal Components Analysis (PCA) see below. In order to select ancestry informative markers (AIMs), we used a previously described procedure in [7, 8] that returns the so-called PCA Informative Markers or PCAIMs for short. The PCAIM selection algorithm uses the geographically significant eigenSNPs (in this case two) and then assigns a score to each SNP. Higher scores correspond to SNPs that correlate well with geography. The algorithm returns the top scoring SNPs, and we have demonstrated that these PCAIMs are very efficient for ancestry prediction [7]. It is worth noting that the method does not take any special measures

in order to avoid redundancy in the set of identified markers. Such redundancy, especially in the case of dense sets of SNP markers, is typically due to tight linkage disequilibrium. In [8] we proposed a linear-algebraic method to remove redundancy from the selected PCAIMs. Our methodology was based on reducing the redundancy removal problem to the so-called Column Subset Selection Problem (CSSP) and on leveraging algorithms and software that are available for the latter problem. This redundancy removal step was employed in our work here.

Encoding the data and handling missing entries

The proportion of missing entries in the POPRES dataset after our quality control step was approximately 2.496%. It is worth emphasizing that all our computations ignore missing entries and thus we do not need to fill in such entries in any manner. We then transformed the raw data to numeric values, without any loss of information, in order to apply our linear algebraic methods. Consider a dataset of a population X consisting of m subjects and assume that for each subject n biallelic SNPs have been assayed. Thus, we are given a table T^X, consisting of m rows and n columns. Each entry in the table is a pair of bases, ordered alphabetically. We transform this initial data table to an integer matrix A^X which consists of m rows – one for each subject – and n columns – one for each SNP. Each entry of A^X will be -1,0, +1, or empty. Let B_1 and B_2 be the bases that appear in the j-th SNP (in alphabetical order). If the genotypic information for the j-th SNP of the i-th individual is B_1B_1 the (i,j)-th entry of A^X is set to +1; else if it is B_1B_2 the (i,j)-th entry of A^X is set to 0; else if it is B_2B_2 the (i,j)-th entry of A^X is set to -1 [7, 8].

The Singular Value Decomposition (SVD) and Principal Components Analysis (PCA)

We briefly describe the Singular Value Decomposition (SVD) of matrices and the related Principal Components Analysis (PCA). Given m subjects and n SNPs, let the $m \times n$ matrix A denote the subject-SNP matrix encoded as described above. After mean-centering the columns (SNP genotypes) of A, the SVD of the matrix returns m pairwise orthonormal vectors u^i, n pairwise orthonormal vectors v^i, and m non-negative singular values σ_i such that $\sigma_1 \geq \sigma_2 \geq \ldots \geq \sigma_m \geq 0$. The matrix A may be written as a sum of outer products as

$$A = \sum_{i=1}^{m} \sigma_i u^i {v^i}^T. \tag{1}$$

Each triplet (σ_i, u^i, v^i) may be used to form a principal component of A. Formally, the i-th most significant principal component of a matrix A is the rank-one matrix that is equal to $\sigma_i u^i {v^i}^T$. In our setting, the left singular vectors (the u^i's) are linear combinations of the columns (SNPs) of A and will be called eigenSNPs [9]. Notice that a principal component is a matrix, whereas an eigenSNP is just a column vector. PCA is a well-known dimensionality reduction technique that, in this case, represents all subjects with respect to a small number of eigenSNPs, corresponding to the top few principal components. All further analysis is then performed on this low-dimensional representation.

Selecting the PCA Informative Markers and removing redundancy

In order to select ancestry informative markers (AIMs), we used a previously described procedure in [7, 8] that returns the so-called PCA Informative Markers or PCAIMs for short and is based on the well-documented fact that PCA reveals population structure [10, 11, 12, 13, 14, 7]. The PCAIM selection algorithm first determines the number of significant principal components (and thus the number of informative eigenSNPs) in the data and then assigns a score to each SNP. In our setting, we are looking to predict an individual's ancestry by predicting his or hers coordinates of origin, and thus we will only use the top two eigenSNPs, since they are clearly correlated with longitude and latitude as shown in prior work [1, 3]. Our methods return SNPs that correlate well with all informative eigenSNPs and we have demonstrated that the selected PCAIMs are very efficient for ancestry prediction [7]. Since the method takes no special measures in order to avoid redundancy in the set of identified markers, we will use the linear-algebraic redundancy removal technique that we proposed in [8]. Our methodology was based on reducing the redundancy removal problem to the so-called Column Subset Selection Problem (CSSP) and on leveraging algorithms and software that are available for the latter problem.

Coordinate prediction via Nearest Neighbors

We model ancestry prediction using panels of AIMs as the following task: given a dataset of m individuals of known coordinates (longitude and latitude) of origin, genotyped on a panel of k AIMs, and a new individual of unknown coordinates of origin genotyped on the same panel, we seek to predict the coordinates of origin of the new sample. This is a standard classification problem and in order to address it we chose to use a simple Nearest Neighbors (NN) approach.

NN-type algorithms first compute the distance of the new sample from the m individuals in the database and then identify the n nearest neighbors of the new sample. In order to predict the coordinates of the new sample, we simply report the average of the coordinates of its n nearest neighbors.

In all our experiments our distance metric was the standard Euclidean (ℓ_2) distance. The distance was computed on the projection of the genotypic data on their top two principal components. We experimented with different values of n (the number of nearest neighbors) ranging from ten up to 20 in increments of one, but we did not observe a consistent advantage in using any value above ten. Thus, we chose to fix n to ten. Similarly, we experimented with various schemes using weighted averages of the coordinates of the top n nearest neighbors (for example, the contribution of the coordinates of a neighbor to the final prediction could be weighted by – some power – of the inverse of its distance to the new sample); once more, we did not observe a consistent advantage in using such schemes. While we can not rule out that more advanced classification methodologies and/or better distance metrics might be applicable in order to improve prediction accuracy, it is quite interesting and exciting that standard, simple methods are quite accurate and useful.

Alternative AIM selection algorithms

It is worth noting that we also experimented with a number of different schemes for selecting AIMs in order to improve our longitude predictions. In particular, we tried selecting only the markers that are directly correlated with the second principal component (ie., the component that is most directly related to longitude). Towards that end, we first selected the 5,000 markers that are most correlated with the second principal component only (note that our PCAIMs are selected using a score that depends on both top two principal components) and then selected panels of 500 and 1000 AIMs using our redundancy removal algorithms. The results were not particularly encouraging: for example, for the TSI crossvalidation experiment, the average error was 4.02 degrees with a standard deviation of 3.964 degrees; this is not considerably better than using the top 1,000 PCAIMs where the error was on average 3.88 degrees with a standard deviation of 4.85 degrees. Keeping 500 SNPs resulted to an average error of 4.66 degrees with a standard deviation of 4.95 degrees, which once more is not a worthwhile improvement.

References

[1] Novembre J, Johnson T, Bryc K, Kutalik Z, Boyko AR, et al. (2008) Genes mirror geography within Europe. Nature 456:98–101.

[2] Nelson MR, Bryc K, King KS, Indap A, Boyko AR, et al. (2008) The Population Reference Sample, POPRES: a resource for population, disease, and pharmacological genetics research. Am J Hum Genet 83:347–358.

[3] Lao O, Lu TT, Nothnagel M, Junge O, Freitag-Wolf S, et al. (2008) Correlation between genetic and geographic structure in Europe. Curr Biol 18:1241–1248.

[4] The International HapMap Consortium (2003) The International HapMap Project. Nature 426:789–796.

[5] The International HapMap Consortium (2005) A haplotype map of the human genome. Nature 437:1299–1320.

[6] Frazer KA, Ballinger DG, Cox DR, Hinds DA, Stuve LL, et al. (2007) A second generation human haplotype map of over 3.1 million SNPs. Nature 449:851–861.

[7] Paschou P, Ziv E, Burchard EG, Choudhry S, Rodriguez-Cintron W, et al. (2007) PCA-correlated SNPs for structure identification in worldwide human populations. PLoS Genet 3:1672–86.

[8] Paschou P, Drineas P, Lewis J, Nievergelt CM, Nickerson DA, et al. (2008) Tracing sub-structure in the European American population with PCA-informative markers. PLoS Genet 4:e1000114.

[9] Lin Z, Altman R (2004) Finding haplotype tagging SNPs by use of principal components analysis. American Journal of Human Genetics 75:850–861.

[10] Menozzi P, Piazza A, Cavalli-Sforza L (1978) Synthetic maps of human gene frequencies in Europeans . Science 201:786–792.

[11] Shriver M, Mei R, Parra E, Sonpar V, Halder I, et al. (2005) Large-scale SNP analysis reveals clustered and continuous patterns of human genetic variation . Hum Genomics 2:81–89.

[12] Patterson N, Price A, Reich D (2006) Population Structure and Eigenanalysis . PLoS Genet 2:e190.

[13] Price A, Patterson N, Plenge R, Weinblatt M, Shadick N, et al. (2006) Principal components analysis corrects for stratification in genome-wide association studies . Nat Genet 38:904–909.

[14] Liu N, Zhao H (2006) A non-parametric approach to population structure inference using multilocus genotypes . Hum Genomics 2:353–364.
