# Supplementary material for: Inferring Geographic Coordinates of Origin for Europeans Using Small Panels of Ancestry Informative Markers
Source: PLoS One. 2010 Aug 18;5(8):e11892. doi: 10.1371/journal.pone.0011892 (PMC2923600; doi:10.1371/journal.pone.0011892)
Supplement: Text S1 — (0.01 MB PDF) [file pone.0011892.s004.pdf]

Online Material

The proposed panels of AIMs are available online at

http://www.cs.rpi.edu/~drinep/POPRESAIMS/
